# Exploring supervised machine learning models to estimate blood pressure using non-fiducial features of the photoplethysmogram (PPG) and its derivatives

**DOI:** 10.1007/s13246-025-01593-3

**Published:** 2025-08-04

**Authors:** Erick Javier Argüello-Prada, Carlos David Castaño Mosquera

**Affiliations:** Programa de Bioingeniería, Facultad de Ingeniería, Universidad Santiago de Cali, Calle 5 # 62-00 Barrio Pampalinda, Cali, 760032 Valle del Cauca Colombia

**Keywords:** Blood pressure, Photoplethysmogram derivatives, Non-fiducial features, Machine learning, Feature selection.

## Abstract

Machine learning has proven valuable in developing photoplethysmography (PPG)-based approaches for blood pressure (BP) estimation, with many holding some promise for cuff-less BP assessment. Still, their efficacy relies on accurate and robust fiducial point detection algorithms. The present study explores the usefulness of several non-fiducial features of the PPG signal and its derivatives in estimating BP by combining well-known feature selection methods and machine learning techniques. We collected PPG recordings from 56 participants and computed fifty-seven non-fiducial features, including statistical indexes and energy operators. After implementing three feature selection algorithms (i.e., *F*-test, mRMR, and ReliefF), the most relevant features were employed to train four learning regression model families. We computed the mean of all absolute errors (MAE), the squared sum and the standard deviation of the errors (MSE and RMSE, respectively), and the coefficient of determination (*r*^2^) to evaluate the performance of each model. The abovementioned feature selection methods produced different optimal feature subsets for systolic and diastolic BP values, with the Matern 5/2 and the rational quadratic GPR models providing the best predictions when combined with ReliefF (MAE = 0.44, MSE = 0.61, and RMSE = 0.78 mmHg for SBP; MAE = 0.31, MSE = 0.40, and RMSE = 0.63 mmHg for DBP). Furthermore, each model utilizes only fifteen easy-to-compute features, thus becoming suitable for computationally constrained hardware. We highlight the need for implementing feature selection algorithms exhaustively, as the most relevant PPG-based features for systolic and diastolic BP estimation might not have the same weight.

## Introduction

High blood pressure (BP), also known as hypertension, is a leading cause of premature death and affects nearly 1.3 billion people worldwide, with an estimated 46% of adults being unaware of having this condition [1, 2]. A possible explanation for such a high prevalence is that hypertension may have little or no symptoms, so it is often referred to as the “silent killer” [3]. Early detection of hypertension is crucial for its prevention and timely treatment. Therefore, there is a need for periodic and reliable measurement of BP.

BP assessment in clinical practice involves using pneumatic cuff-based devices, among which the mercury sphygmomanometer is still the standard for assessing BP without the constraints of invasive procedures like catheterization. On the other hand, sphygmomanometers are unsuitable for continuous monitoring because of cuff discomfort and the extended interval needed to obtain BP measurements [4]. In this sense, there has been intensive research in developing and testing cuff-less approaches for measuring BP. Photoplethysmography (PPG) has emerged as a promising alternative to estimate BP due to its non-invasive character, portability, and low cost. PPG employs one optical sensor to capture red or infrared light absorption changes caused by the wave-like motion of the blood through the arteries, giving rise to a pulsatile waveform known as the PPG signal [5]. More prevalent methods include those employing time- and frequency-domain features of the PPG signal and its derivatives. In the literature, the first derivative of the PPG signal is also known as the velocity plethysmogram (VPG), whereas the second derivative is often called the acceleration plethysmogram (APG) [6]. Given the outstanding potential of machine-learning algorithms to process large volumes of data, several authors have used these features to train machine-learning models for cuff-less BP assessment [7, 8]. These studies can either differentiate normotensive from pre-hypertensive and hypertensive individuals [9–11] or provide a numerical estimate of BP using classification or regression learning models [12–14].

When developing classification models for BP assessment, researchers utilize the metrics proposed by the Association for Advancement of Medical Instrumentation (AAMI) for electrocardiography (ECG)-based detection algorithms [15], such as sensitivity, precision, and accuracy, to evaluate the model’s performance. On the other hand, the root-squared mean error (RMSE), the mean absolute error (MAE), and the correlation coefficient (*r*) are the most commonly employed metrics for evaluating the performance of regression models for BP estimation. Using publicly available datasets to validate novel vital signs estimation methods can provide comparable results and allow for reproducibility. In the context of BP estimation through features extracted from the PPG signal, most authors have used the Physionet’s Multi-Parameters Intelligent Monitoring in Intensive Care Units (MIMIC), which contains ECG, PPG, and ABP signals simultaneously recorded from Intensive Care Unit (ICU) patients [16]. However, diverse pathophysiological conditions and medication or sedative drugs administered to ICU patients may affect PPG signals’ contour and, thus, their relationship to BP. Therefore, results provided by approaches tested on this dataset can hardly be extrapolated to healthy subjects, and their applicability to other cohorts requires further investigation on external datasets [17]. Moreover, there is always a risk of overlapping patients between older and newer versions of the MIMIC database (e.g., MIMIC III and MIMIC IV).

Most studies on BP assessment rely on fiducial point detection algorithms to extract several features from the PPG signal and its derivatives. For example, Gupta and co-workers [12] used systolic and diastolic peak amplitudes as well as global maxima and minima of VPG and APG signals, respectively, to compute the mean arterial pressure (MAP) by training three popular regressors: random forest, XGBoost, and Elastic net (MIMIC I: MAE = 2.93 mmHg and *r* = 0.82; MIMIC II: MAE = 1.28 mmHg and *r* = 0.92). Aguet and colleagues [18] achieved an error (mean ± standard deviation) of − 0.87 ± 10.77 mmHg for systolic blood pressure (SBP) and − 1.31 ± 7.62 mmHg for diastolic blood pressure (DBP) combining features derived from the PPG first, second, and third derivatives with Lasso (least absolute shrinkage and selection operator) regression. More recently, Nishan and collaborators [19] used time- and frequency-domain features extracted from PPG, VPG, and APG signals to train four regressors, among which the support vector regressor (SVR) outperformed other algorithms in estimating the SBP, DBP, and MAP (MAE = 2.49, 1.43, and 1.62 mmHg, respectively). While promising, the results reported by these and several other studies [20–23] are limited by the accuracy of the fiducial detection method used. VPG and APG fiducial points (e.g., the diastolic peak amplitude of VPG) are not always clearly identifiable due to the high variability in the morphology of the PPG signal [24]. Failures in detecting PPG, VPG, and APG fiducial points may compromise feature computation and lead to erroneous conclusions when estimating BP. On the other hand, non-fiducial features can reveal discriminative information from the signal without requiring fiducial point identification. Furthermore, non-fiducial analysis is often less computationally expensive than fiducial analysis, as the former does not involve the computational costs associated with fiducial point detection methods. Therefore, the present study aims to enhance BP estimation by combining non-fiducial features from PPG, VPG, and APG waveforms with supervised machine learning techniques. Main contributions of this work are outlined below:

- Unlike previous research on this topic [25–28], an extensive exploration of non-fiducial features of PPG, VPG, and APG signals was conducted to ensure the most relevant in estimating BP.
- A new dataset containing PPG records of 56 subjects is created and made publicly available to validate PPG-based BP assessment approaches. We did not recruit critically ill or hospitalized subjects to minimize the potential effects of diverse pathophysiological conditions and medications on PPG waveforms, as they may reduce the predictive power of the models for the general population [17].

The rest of this article is organized as follows. Section “Methods” contains some considerations about the selection of machine learning models for PPG-based estimation of BP. The experimental protocol, signal processing, feature extraction methods, and machine learning algorithms are detailed in Section “Results”. Sections “Discussion”and “Conclusion” present the results and discussion, respectively.

## Methods

### Data acquisition module

**Fig. 1:**
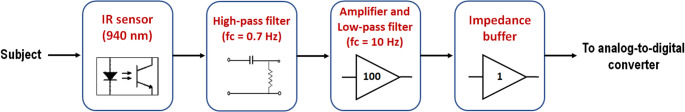
Schematic diagram of the PPG signal acquisition module

We built a reflectance-PPG probe by embedding a TSAL6400 near-infrared (NIR) light-emitting diode (LED) and a TEMD5080 × 01 NIR photodiode (Vishay Semiconductors, USA) into a NellcorTM adult finger clip (model DS-100 A) to collect peripheral blood volume changes from the participants. The TSAL6400 NIR-LED has a peak emission wavelength of 940 nm at a forward current of 100 mA, achieving good spectral matching with the TEMD5080 × 01 photodetectors, whose peak sensitivity is also 940 nm. As shown in Fig. 1, a passive high-pass filter (cutoff frequency = 0.7 Hz) and an active low-pass filter (cutoff frequency = 10 Hz; gain = 100) were combined to band-pass the photodetector’s output signal. According to Campbell and colleagues [29], an acceptable frequency band for band-pass filtering the PPG signal is between 0.66 Hz and 4.5 Hz, which covers the physiological heart rate range (i.e., 40 and 270 beats per minute). Nevertheless, we extended the low-pass cutoff frequency to 10 Hz as the frequency components of the dicrotic notch are twice the heart rate [29]. A second amplifier in a buffer configuration (gain = 1) was added to enable offset control and prevent signal saturation. A 10-bit resolution analog-to-digital converter of an Arduino Nano board (Arduino LLC, USA) digitalized the PPG signal at a sampling rate of 100 Sa/s, which was transmitted to a laptop via a USB 2.0 link for offline processing.

### Subjects and data collection

Fifty-six volunteers (22 males and 34 females; mean age ± standard deviation = 52.5 ± 7.2 years) participated in the study. No subject had a previous history of suffering from diabetes, cardio-respiratory diseases, or neurological disorders, and all followed abstinence from drinking and smoking for three hours before the experiment. Written informed consent was obtained from all subjects, who were previously fully informed about the experimental protocol.

Data were collected after the approval of the Ethics Committee of the Universidad Santiago de Cali’s Engineering Faculty (registry code 088) and in compliance with the Declaration of Helsinki. Each participant arrived at the laboratory and was seated in a backrest chair as comfortably as possible, with both arms flat on a front desktop. The room temperature was at 22.5 °C and only one researcher and one volunteer were present during each session for data collection. After five minutes, we instructed each subject to place the right index finger on the PPG probe while attaching a cuff-based electronic sphygmomanometer (GlucoQuick P30 Plus) to their left forearms. PPG and BP data were obtained within 3 min, while volunteers remained quiet and motionless. The PPG recording period lasted 2 min, and BP was measured before acquiring PPG data. We saved PPG records with no personal identifiers as MAT files into the MATLAB programming environment (version 2021b, the Mathworks Inc., Natick, USA). Subjects’ SBP and DBP values ranged from 97 to 164 mmHg and from 57 to 109 mmHg, respectively. Table 1 summarizes the participants’ demographics according to the American Heart Association [30].

**Table 1:** Blood pressure characteristics of the sample

Total data sample	56 (100%)
Subjects with normal BP	17 (30%)
Subjects with elevated BP	11 (20%)
Subjects with stage 1 hypertension	14 (25%)
Subjects with stage 2 hypertension	14 (25%)

### Signal processing and feature extraction

**Fig. 2:**
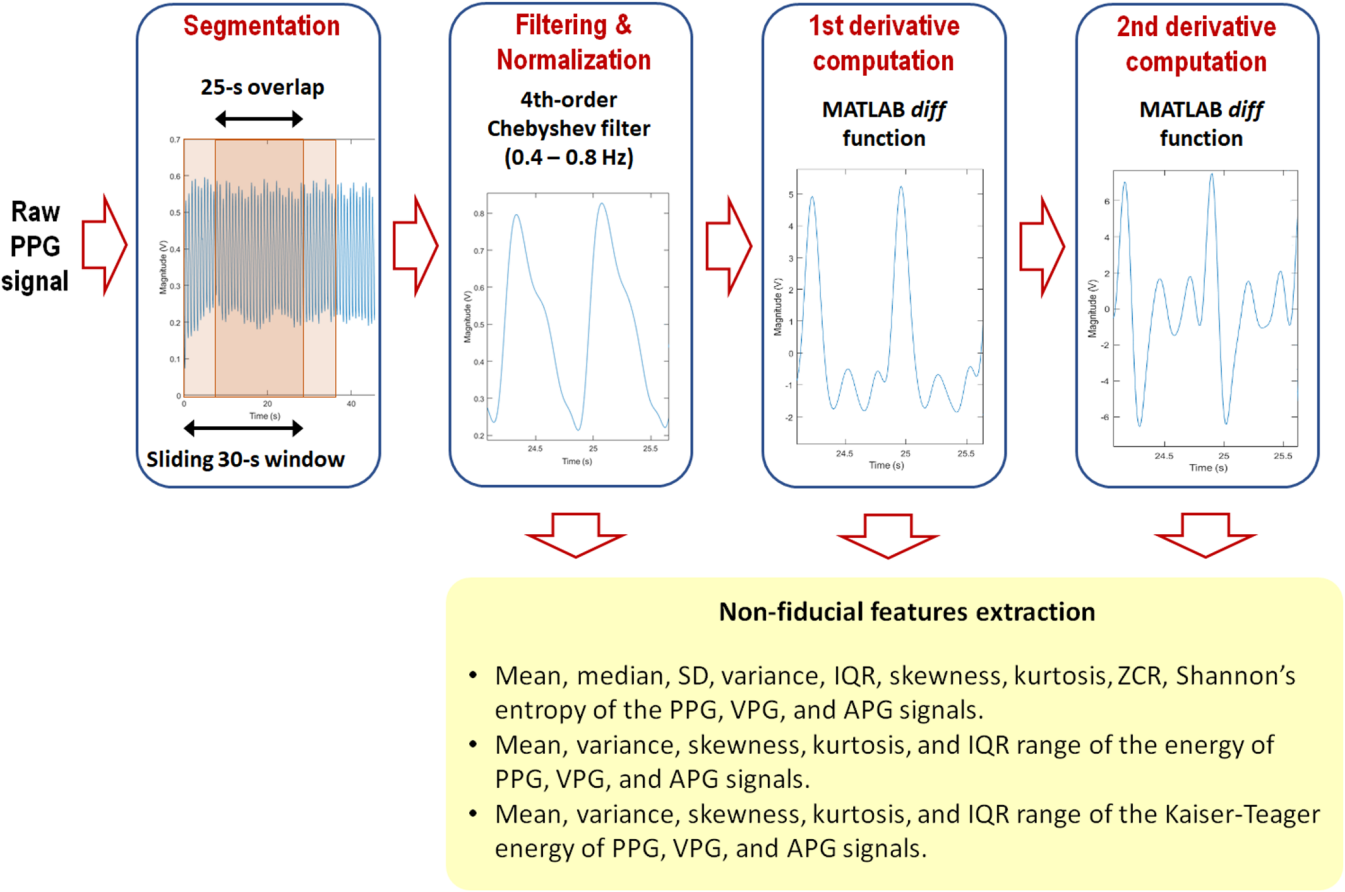
A flowchart of the signal processing and feature extraction stages. APG: Acceleration plethysmogram; IQR: interquartile range; PPG: photoplethysmogram; SD: standard deviation; VPG: velocity plethysmogram; ZCR: zero crossing rate

The recorded PPG signals were segmented using overlapped windowing (window size of 30 s with 25 s of overlap), as shown in Fig. 2. PPG segments’ quality was improved using a 4th-order Chebyshev type II filter (0.4–8 Hz), as recommended by Liang and colleagues [31]. After normalizing PPG waveforms, we computed VPG and APG via the MATLAB *diff* function and applied a Savitzky-Golay filter to minimize the random noise. Nineteen non-fiducial features (see Table 2) were extracted from PPG, VPG and APG signals and exported to a predesigned Excel spreadsheet. We first considered including statistical features as they are relatively easy to calculate. After a previous literature review, we found that statistical features, such as the variance, skewness, kurtosis, zero crossing rate (ZCR), and Shannon’s entropy of the PPG signal, have proven valuable in developing machine learning-based approaches for BP estimation [25–28]. However, the utilization of several other statistical features like the signal’s mean or median has not been previously reported. The mean of the PPG signal reflects the strength of its non-pulsatile component, which has been shown to correlate with MAP values [32]. Therefore, we added the mean, median, standard deviation, and interquartile range to provide a more comprehensive set of statistical features.

Other non-fiducial features like energy operators have also been reported in the context of BP estimation. For instance, Monte-Moreno employed several metrics derived from the Kaiser–Teager energy (KTE) of the PPG signal [27]. KTE is a standard tool for identifying energy profiles of periodic signals, and it has proven helpful in separating noise or artifacts and transients from the signal [33]. On the other hand, it focuses on instantaneous energy values, providing a local measure of the signal’s energy. Global measures of the energy of the PPG signal have also been considered for estimating BP [28]. However, no study seems to have reported the potential of the metrics that can be derived from the signal’s energy. Thus, we expanded our feature set by including various metrics derived from the energy and KTE of the PPG, VPG, and APG waveforms. Non-fiducial features like indexes extracted from Poincaré plots have also been identified in the context of BP estimation from PPG signals [34]. Still, they have not been included in the present study to not increase the complexity of the proposed approach.

**Table 2:** List of features used in this study

Feature description	Notation	Reference work
*Signal mean*: the average value of the signal, also viewed as its non-pulsatile (DC) component	$\mu\left(x\right)$	Not reported
*Signal median*: the value of the signal with an equal number of values above and below it	η$\left(x\right)$	Not reported
*Signal standard deviation*: describes how far apart the signal values are from the signal mean	$\sigma\left(x\right)$	Not reported
*Signal variance*: describes the degree of dispersion of signal values or how much the signal values vary between them	${\sigma}^{2}\left(x\right)$	[25]
*Signal interquartile range*: expresses the variation of the signal values by dividing the total value range into quartiles	$IQR\left(x\right)$	Not reported
*Signal skewness*: provides a representation of the degree of asymmetry in the signal values distribution	$skew\left(x\right)$	[25, 26]
*Signal kurtosis*: describes the shape of the signal values distribution (peaked or flat)	$kurt\left(x\right)$	[25, 26]
*Zero crossing rate*: denotes the change rate of the signal’s sign	$ZCR\left(x\right)$	[27]
*Shannon’s entropy*: provides a measure of the signal’s predictability	$H\left(x\right)$	[27, 28]
*Energy mean*: the mean of the signal’s energy	${E}_{\mu}\left(x\right)$	[28]
*Energy variance*: the variance of the signal’s energy	${E}_{{\sigma}^{2}}\left(x\right)$	Not reported
*Energy skewness*: the skewness of the signal’s energy	${E}_{skew}\left(x\right)$	Not reported
*Energy kurtosis*: the kurtosis of the signal’s energy	${E}_{kurt}\left(x\right)$	Not reported
*Energy interquartile range*: the interquartile of the signal’s energy	${E}_{IQR}\left(x\right)$	Not reported
*Kaiser–Teager energy mean*: the mean of the signal’s Kaiser–Teager energy	${KTE}_{\mu}\left(x\right)$	[27]
*Kaiser–Teager energy variance*: the variance of the signal’s Kaiser–Teager energy	${KTE}_{{\sigma}^{2}}\left(x\right)$	[27]
*Kaiser–Teager energy skewness*: the skewness of the signal’s Kaiser–Teager energy	${KTE}_{skew}\left(x\right)$	[27]
*Kaiser–Teager energy kurtosis*: the kurtosis of the signal Kaiser–Teager energy	${KTE}_{kurt}\left(x\right)$	Not reported
*Kaiser–Teager energy interquartile range*: the interquartile range of the signal’s Kaiser–Teager energy	${KTE}_{IQR}\left(x\right)$	[27]

### Feature selection

While we computed 57 features, we did not consider all of them to address the problem posed in this study. Too many features may increase complexity and deteriorate the learning models’ performance, so examining the dependency of the target variable with each computed feature and, in turn, removing the redundant ones is crucial to building a more comprehensive model [35]. Like machine learning models, diverse feature selection methods have been applied to BP estimation without a clear preference for any specific technique [8, 36]. As a result, we employed three well-known feature selection algorithms based on their differing approaches to identifying the most relevant features: the one-way analysis of variance (ANOVA) *F*-test [37], minimum redundancy-maximum relevance (mRMR) [38], and ReliefF [39]. The *F*-test selects features for which the variance of sample points within the same class is minimal, while the variance of sample points across different classes is maximal. However, this test does not account for feature redundancy, unlike the mRMR technique, which selects features that exhibit both minimal correlation with each other and high correlation with the output variable. In contrast, ReliefF evaluates the quality of features by assessing their ability to differentiate between pairs of nearest-neighbor instances.

### Selection of machine learning methods for estimating blood pressure from PPG features

Machine learning algorithms have brought notable advancements in PPG-based estimation of BP, and authors have used diverse learning models for this purpose. According to recent literature reviews [8, 40], linear regressions (LRs), artificial neural networks (ANNs), and support vector machines (SVMs) have often been used to estimate BP from PPG features. However, several other machine-learning models, such as regression trees (RTs) and Gaussian process regressions (GPRs), have also provided promising results for cuff-less BP prediction [18, 19, 22, 41]. In many studies, authors train and test different learning models to select the one with greater predictive accuracy (i.e., flexibility). But as important as the model’s accuracy is, its interpretability (i.e., the degree to which someone can explain how the model arrived at the prediction) is just as important. The relationship between these two concepts often presents as a trade-off [42]. For instance, linear regressions are highly interpretable and fast for making predictions. Still, they usually have low predictive accuracy due to their highly constrained form. On the contrary, models capable of achieving greater accuracy (e.g., random forests) are often not tractable and do not allow for an explanation of how or why they produced a specific result [43].

Without a clear preference for a specific machine learning algorithm [36], it is crucial to consider the model’s predictive accuracy and interpretability. Therefore, we selected four model families with multiple variants offering different predictive accuracy and interpretability levels:

#### Linear regressions (LRs)

LRs describe the relationship between a dependent variable and one or more independent variables (i.e., predictors). In its simplest form, the model is given by Eq. (1), where *y* is the predicted variable, *x*_*i*_ is the *i*-th predictor, β_i_ is the coefficient of the *i*-th predictor, and ϵ represents the fixed error of the model [44]:

1 $$y={\beta}_{0}+{\beta}_{1}{x}_{1}+{\beta}_{2}{x}_{2}+\cdots\,{\beta}_{n}{x}_{n}+\epsilon.$$

Simple linear, interaction linear, robust linear, and stepwise linear regressions were considered in this study.

#### Regression trees (RTs)

RTs are decision trees for which the predicted variables can take continuous values instead of class labels in leaves [45]. They are easy to interpret and low on memory use. As the model flexibility depends on the leaves’ size and number, we used coarse, medium, and fine trees.

#### Support vector regressors (SVRs)

SVRs use kernels, such as radial basis function (RBF), to reflect nonlinear relationships and find the optimal hyperplane [46]. The input feature set, *F* = {*f*_1_, *f*_2_,…, *f*_n_}, is first mapped to a high-dimensional space through *m* nonlinear transformations denoted as *φ*_*i*_() to give a prediction according to Eq. (2), where *ω*_*i*_ is the coefficient of the *i*-th regression function and *b* is the prediction bias:

2 $$y=\sum_{i=1}^{m}{\omega}_{i}{\phi}_{i}\left(F\right)+b.$$

For this study, linear, quadratic, cubic, fine Gaussian, medium Gaussian, and coarse Gaussian SVRs were considered.

#### Gaussian process regressions (GPRs)

The GPR family encompasses all non-parametric optimization functions, which, unlike other supervised learning regression models, use a probabilistic (i.e., Bayesian) approach to infer a probability distribution over all possible values, thus capturing the model’s uncertainty [47]. They are highly flexible but difficult to interpret. Bayes’ rule is expressed by Eq. (3), where *p*(*y*|*x*) is the marginal probability of the data, *p*(*f*) is the prior distribution of the function *f*, and p(*y*|*x*,*f*) is the likelihood of the data given *f*:

3 $$p\left(f|x,y\right)=\frac{p\left(y|x,f\right)p\left(f\right)}{p\left(y|x\right)}.$$

Rational quadratic, squared exponential, Matern 5/2, and exponential GPRs were considered in this research.

All the previously described models were trained and tested on the optimal subset selected by the abovementioned feature selection techniques. We split the data into training and validation sets using a stratified K-fold cross-validation approach (K = 10) to preserve the proportion of samples of each class across folds.

### Performance assessment

Knowing how close the predicted values are to the expected ones is crucial to assessing regression models’ performance. Error metrics can summarize how large the differences between predictions and ground truth values are on average, so they have been widely used in machine learning research to report regression models’ performance. For this study, we computed the average of the absolute differences between predicted and reference values (see Eq. (4)), commonly known as the mean absolute error (MAE), the average of the squared differences and the square root of the mean squared differences (see Eqs. (5) and (6)), also referred to as the mean squared error (MSE) and the root mean squared error (RMSE), respectively. In Eqs. (4)–(6), *BP*_*p*_ and *BP* denote the predicted and expected BP values, and *N* represents the total number of paired observations. We also calculated the coefficient of determination (*r*^2^), which expresses the percentage of variability in one measurement that is accounted for by the other (see Eqs. (7) and (8)). Note that *MSE*(*baseline*) in Eq. (8) represents the variance of the BP actual values as given by the squared difference between actual values and the total mean of the actual values:

4 $$MAE=\frac{1}{N}\sum\limits_{i=1}^{N}\left|{BP}_{p}\left(i\right)-BP\left(i\right)\right|,$$

5 $$MSE=\frac{{\sum}_{i=1}^{N}{\left({BP}_{p}\left(i\right)-BP\left(i\right)\right)}^{2}}{N},$$

6 $$RMSE=\sqrt{MSE},$$

7 $${r}^{2}=1-\frac{MSE\left(model\right)}{MSE\left(baseline\right)},$$

8 $$MSE\left(baseline\right)=\frac{{\sum}_{i=1}^{N}{\left(BP\left(i\right)-mean\left(BP\right)\right)}^{2}}{N},$$

Bland–Altman plots have proven valuable in assessing the agreement and interchangeability between standardized and proposed approaches. As estimating precision depends on the amount of observed data (i.e., the sample size), it would be convenient to calculate the confidence interval (CI) to determine how precise our estimates are [48]. Thus, we computed the 95% CI of the mean difference between predicted and reference BP values to describe the extent of the systematic difference.

## Results

As shown in Table 3, the three feature selection methods described above yielded different optimal subsets of fifteen features for each target variable (i.e., SBP and DBP).

**Table 3:** The top 15 features according to *F*-test, mRMR, and relieff, from the most to the less relevant

Relevance	*F*-test		mRMR		ReliefF	
	SBP	DBP	SBP	DBP	SBP	DBP
1	${E}_{{\sigma}^{2}}\left(APG\right)$	${E}_{kurt}\left(APG\right)$	$\mu\left(PPG\right)$	$\mu\left(PPG\right)$	${E}_{IQR}\left(PPG\right)$	${KTE}_{IQR}\left(PPG\right)$
2	${E}_{{\sigma}^{2}}\left(VPG\right)$	${E}_{{\sigma}^{2}}\left(VPG\right)$	${E}_{IQR}\left(APG\right)$	${KTE}_{\mu}\left(APG\right)$	${KTE}_{IQR}\left(PPG\right)$	${E}_{IQR}\left(PPG\right)$
3	${KTE}_{\mu}\left(VPG\right)$	$skew\left(APG\right)$	$kurt\left(PPG\right)$	$skew\left(PPG\right)$	$skew\left(APG\right)$	$skew\left(APG\right)$
4	${KTE}_{{\sigma}^{2}}\left(VPG\right)$	${E}_{skew}\left(APG\right)$	${KTE}_{kurt}\left(PPG\right)$	${E}_{skew}\left(PPG\right)$	$IQR\left(APG\right)$	$skew\left(PPG\right)$
5	${E}_{\mu}\left(APG\right)$	${E}_{{\sigma}^{2}}\left(APG\right)$	$ZCR\left(APG\right)$	$H\left(VPG\right)$	η$\left(APG\right)$	$ZCR\left(VPG\right)$
6	${KTE}_{\mu}\left(APG\right)$	$H\left(VPG\right)$	$skew\left(APG\right)$	${KTE}_{kurt}\left(VPG\right)$	$ZCR\left(VPG\right)$	$\eta\left(APG\right)$
7	${E}_{kurt}\left(APG\right)$	${KTE}_{\mu}\left(VPG\right)$	${E}_{skew}\left(PPG\right)$	$ZCR\left(APG\right)$	$skew\left(VPG\right)$	${E}_{{\sigma}^{2}}\left(VPG\right)$
8	${E}_{skew}\left(APG\right)$	${E}_{\mu}\left(APG\right)$	$IQR\left(APG\right)$	$IQR\left(VPG\right)$	$skew\left(PPG\right)$	$IQR\left(APG\right)$
9	$\sigma\left(APG\right)$	${KTE}_{{\sigma}^{2}}\left(VPG\right)$	${E}_{IQR}\left(PPG\right)$	${E}_{IQR}\left(PPG\right)$	$ZCR\left(APG\right)$	$H\left(PPG\right)$
10	${\sigma}^{2}\left(APG\right)$	${KTE}_{\mu}\left(APG\right)$	$H\left(VPG\right)$	${KTE}_{kurt}\left(PPG\right)$	η$\left(PPG\right)$	${E}_{{\sigma}^{2}}\left(APG\right)$
11	${E}_{\mu}\left(VPG\right)$	${KTE}_{IQR}\left(APG\right)$	$H\left(PPG\right)$	η$\left(APG\right)$	$H\left(APG\right)$	$skew\left(VPG\right)$
12	${KTE}_{IQR}\left(APG\right)$	${E}_{IQR}\left(VPG\right)$	η$\left(PPG\right)$	η$\left(PPG\right)$	$IQR\left(PPG\right)$	$kurt\left(PPG\right)$
13	${KTE}_{kurt}\left(APG\right)$	${E}_{\mu}\left(VPG\right)$	${KTE}_{kurt}\left(APG\right)$	$IQR\left(APG\right)$	$H\left(PPG\right)$	${E}_{IQR}\left(VPG\right)$
14	${KTE}_{IQR}\left(VPG\right)$	$\sigma\left(APG\right)$	$skew\left(VPG\right)$	${KTE}_{kurt}\left(APG\right)$	$\mu\left(PPG\right)$	$\eta\left(PPG\right)$
15	${E}_{IQR}\left(APG\right)$	${\sigma}^{2}\left(APG\right)$	${E}_{{\sigma}^{2}}\left(APG\right)$	${E}_{{\sigma}^{2}}\left(PPG\right)$	$\sigma\left(PPG\right)$	$IQR\left(VPG\right)$

Tables 4, 5 and 6 show that GPR models outperform LRs, RTs, and SVRs as they achieve minimal errors and the uppermost coefficients of determination. In particular, the Matern 5/2 Gaussian process regression, combined with the ReliefF feature selection method, provides the lowest errors (MAE = 0.44, MSE = 0.61, and RMSE = 0.78 mmHg) and the highest coefficient of determination (*r*^2^ = 1) for SBP. On the other hand, the rational quadratic Gaussian process regression achieves the lowest errors (MAE = 0.31, MSE = 0.40, and RMSE = 0.63 mmHg) and the highest coefficient of determination (*r*^2^ = 1) for DBP when combined with ReliefF.

**Table 4:** Performance of the regression learning models using the *F*-test feature selection method (errors reported in mmHg)

	SBP						DBP					
	RMSE	*R* ^2^	MSE	MAE	Prediction speed (obs/s)	Training time (s)	RMSE	*R* ^2^	MSE	MAE	Prediction speed (obs/s)	Training time (s)
Simple linear	12.07	0.33	145.69	9.10	6500	8.8992	9.56	0.24	91.30	6.98	17,000	3.0498
Interactions linear	24.60	− 1.80	604.99	9.12	2800	11.694	7.98	0.47	63.74	4.77	4400	4.234
Robust linear	12.28	0.30	150.88	9.13	11,000	11.315	10.22	0.13	104.42	7.02	18,000	3.9704
Step-wise linear	11.04	0.44	121.91	8.05	10,000	861.37	7.49	0.53	56.08	4.63	12,000	1122.1
Fine tree	7.59	0.73	57.56	4.04	8300	12.403	5.60	0.74	31.37	2.39	35,000	4.6579
Medium tree	7.90	0.71	62.35	5.15	25,000	12.98	6.22	0.68	38.68	3.47	35,000	5.3778
Coarse tree	9.36	0.60	87.55	7.07	28,000	12.721	7.10	0.58	50.42	4.97	34,000	5.18
Linear SVR	12.32	0.30	151.76	9.01	15,000	16.429	9.89	0.19	97.83	6.76	35,000	8.0684
Quadratic SVR	18.29	− 0.55	334.48	8.14	34,000	203.08	6.70	0.63	44.94	4.29	33,000	77.016
Cubic SVR	193.00	− 171.21	37259.00	21.23	21,000	559.19	90.91	− 67.74	8264.90	6.32	22,000	396.98
Fine Gaussian SVR	7.80	0.72	60.85	4.97	21,000	560	3.19	0.92	10.21	1.88	24,000	398.11
Medium Gaussian SVR	11.85	0.35	140.50	8.22	14,000	561.09	6.59	0.64	43.46	4.23	18,000	398.95
Coarse Gaussian SVR	12.55	0.27	157.51	9.50	14,000	561.92	9.71	0.22	94.22	6.47	18,000	399.9
Squared exponential GPR	5.66	0.85	32.02	3.09	7800	673.36	2.63	0.94	6.96	1.17	19,000	455.98
Matern 5/2 GPR	5.16	0.88	26.58	2.73	15,000	724.27	2.14	0.96	4.59	0.97	14,000	558.38
Exponential GPR	5.16	0.88	26.62	2.91	14,000	797.74	2.22	0.96	4.91	1.18	18,000	624.3
Rational quadratic GPR	**5.02**	**0.88**	**25.15**	**2.60**	**10,000**	**919.26**	**1.96**	**0.97**	**3.85**	**0.93**	**8600**	**844.13**

**Table 5:** Performance of the regression learning models using the mRMR feature selection method (errors reported in mmHg)

	SBP						DBP					
	RMSE	*R* ^2^	MSE	MAE	Prediction speed (obs/s)	Training time (s)	RMSE	*R* ^2^	MSE	MAE	Prediction speed (obs/s)	Training time (s)
Simple linear	11.79	0.36	139.02	9.07	20,000	2.6301	9.20	0.29	84.57	6.91	17,000	3.1566
Interactions linear	7.10	0.77	50.36	4.69	2800	2.6862	8.21	0.44	67.44	4.64	3000	6.7768
Robust linear	12.33	0.30	152.10	8.53	12,000	4.2066	10.01	0.16	100.16	6.78	11,000	6.4391
Step-wise linear	6.63	0.80	44.01	4.78	8000	1620.2	6.24	0.67	38.95	4.45	9200	1144.8
Fine tree	4.79	0.89	22.96	1.81	29,000	5.6783	3.97	0.87	15.75	1.35	35,000	5.7177
Medium tree	5.32	0.87	28.25	2.90	34,000	5.3997	4.54	0.83	20.61	2.23	30,000	8.2198
Coarse tree	7.80	0.72	60.79	5.79	40,000	4.9698	6.78	0.62	45.95	4.80	23,000	7.802
Linear SVR	12.33	0.30	152.07	8.37	32,000	7.1345	9.47	0.25	89.70	6.50	19,000	8.9201
Quadratic SVR	6.55	0.80	42.95	4.14	34,000	28.125	6.09	0.69	37.09	3.76	31,000	25.179
Cubic SVR	21.36	− 1.11	456.40	3.45	19,000	158.64	8.42	0.41	70.91	3.38	28,000	110.14
Fine Gaussian SVR	3.31	0.95	10.98	1.82	25,000	159.21	3.60	0.89	12.97	2.06	26,000	111.11
Medium Gaussian SVR	6.16	0.82	37.99	3.78	20,000	159.62	4.94	0.80	24.43	3.10	23,000	111.73
Coarse Gaussian SVR	11.74	0.36	137.72	7.79	23,000	160.48	8.80	0.35	77.39	5.84	21,000	112.42
Squared exponential GPR	2.01	0.98	4.05	0.79	15,000	265.93	2.67	0.94	7.12	1.14	22,000	188.59
Matern 5/2 GPR	1.75	0.99	3.05	0.69	17,000	349.37	2.32	0.95	5.40	0.96	17,000	277.47
Exponential GPR	1.95	0.98	3.81	0.95	21,000	418.82	2.41	0.95	5.79	1.15	22,000	351.37
Rational quadratic GPR	**1.73**	**0.99**	**2.99**	**0.69**	**9400**	**616.14**	**2.25**	**0.96**	**5.05**	**0.92**	**11,000**	**551.58**

**Table 6:** Performance of the regression learning models using the relieff feature selection method (errors reported in mmHg)

	SBP						DBP					
	RMSE	*R* ^2^	MSE	MAE	Prediction speed (obs/s)	Training time (s)	RMSE	*R* ^2^	MSE	MAE	Prediction speed (obs/s)	Training time (s)
Simple linear	11.33	0.41	128.32	8.53	14,000	2.9017	8.89	0.34	79.09	6.61	6600	11.4
Interactions linear	5.41	0.86	29.29	4.13	3900	5.7097	4.20	0.85	17.62	2.70	2000	17.499
Robust linear	12.25	0.31	150.01	8.38	13,000	5.3027	9.29	0.28	86.28	6.48	12,000	16.823
Step-wise linear	5.65	0.85	31.92	4.30	9200	1741.3	3.79	0.88	14.39	2.80	8200	1958.4
Fine tree	4.83	0.89	23.30	1.55	38,000	4.4804	4.10	0.86	16.83	1.41	15,000	15.226
Medium tree	6.23	0.82	38.79	3.05	38,000	7.4078	5.08	0.79	25.76	2.22	28,000	23.538
Coarse tree	9.03	0.62	81.54	6.30	38,000	6.7416	6.53	0.64	42.63	4.15	19,000	22.08
Linear SVR	11.88	0.35	141.23	8.22	27,000	8.6056	9.18	0.30	84.23	6.13	11,000	19.302
Quadratic SVR	6.13	0.83	37.57	3.77	37,000	28.727	3.78	0.88	14.27	2.50	23,000	81.229
Cubic SVR	2.54	0.97	6.43	1.67	32,000	322.28	2.37	0.91	11.18	1.36	26,000	304.23
Fine Gaussian SVR	2.11	0.98	4.47	1.43	23,000	323.15	2.37	0.95	5.61	1.46	21,000	305.15
Medium Gaussian SVR	4.87	0.89	23.68	2.99	23,000	323.97	2.85	0.93	8.13	1.90	23,000	306.23
Coarse Gaussian SVR	11.55	0.38	133.36	7.87	21,000	324.72	8.59	0.38	73.81	5.64	19,000	307.37
Squared exponential GPR	0.82	1.00	0.67	0.47	21,000	395.76	0.78	0.99	0.60	0.36	14,000	410.54
Matern 5/2 GPR	**0.78**	**1.00**	**0.61**	**0.44**	**16,000**	**496.04**	0.65	1.00	0.42	0.32	15,000	513.07
Exponential GPR	1.18	0.99	1.38	0.70	15,000	571.93	0.97	0.99	0.94	0.51	16,000	586.53
Rational quadratic GPR	0.79	1.00	0.63	0.44	10,000	811.23	**0.63**	**1.00**	**0.40**	**0.31**	**9600**	**820.18**

**Fig. 3:**
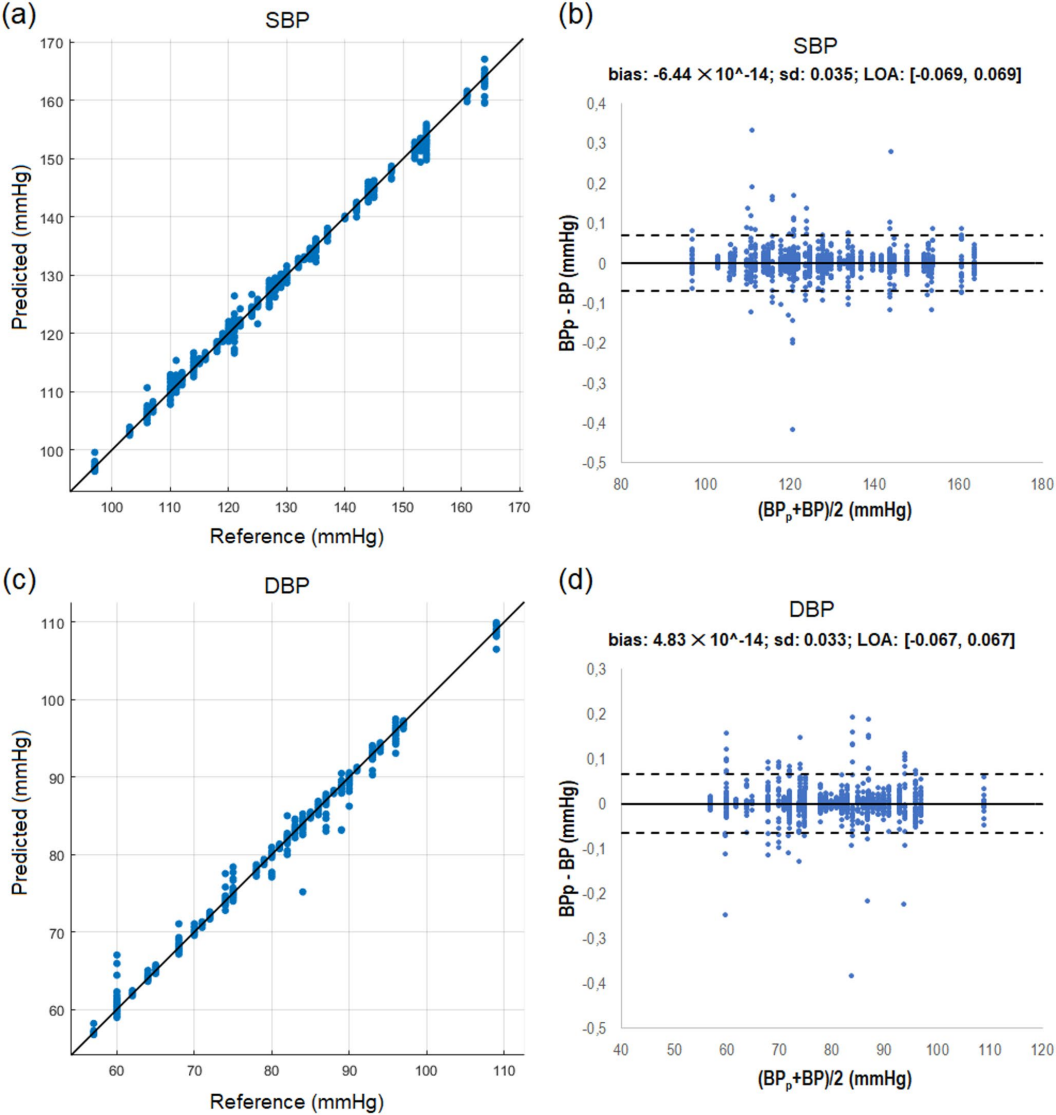
graphical representation of the agreement between predicted and reference values (*n* = 1120) for the Matern 5/2 GPR model (SBP) and the rational quadratic GPR model (DBP): **a** SBP regression plot; **b** SBP Bland–Altman plot; **c** SBP regression plot; **d** DBP Bland–Altman plot. The bias and the limits of agreement (bias ± 1.96 × standard deviation) are shown by bold solid and dashed lines, respectively

Figure 3 presents scatter plots illustrating the relationship between predicted and actual BP values (regression plots, panels (a) and (c)), as well as the difference between these two measures compared to their average (Bland–Altman plots, panels (b) and (d)), for the most effective prediction models (Matern 5/2 GPR for SBP and rational quadratic GPR for DBP). As indicated in Table 7, the 95% CI of the mean difference between predicted and reference BP values of both models contained the line of equality (i.e., *BP*_*p*_ – *BP* = 0).

**Table 7:** 95% Confidence intervals (CI) of the differences between predicted and reference BP values

BP estimation	95% CI	
	From	To
SBP (Matern 5/2 GPR)	− 0.0021	0.0021
DBP (rational quadratic GPR)	− 0.0020	0.0020

## Discussion

Results show that Gaussian process regression (GPR) models produced the best BP estimates when combined with the ReliefF-based feature selection method. The same combination yielded the best prediction results when used by Chowdhury and colleagues [22], although they did not consider non-fiducial features from PPG signals and their derivatives. Instead, they employed several amplitude-, time-, and frequency-domain features combined with demographic data, such as age and body mass index (BMI). In this study, GPR models achieved the lowest errors and the highest coefficients of determination compared to LR, RT, and SVR families, regardless of the feature selection technique (see Tables 4, 5 and 6). However, they also showed the highest training times, which could be impractical for applications with limited computational resources. Despite their probabilistic approach, GPR models are more complex and less tractable than traditional regression models, so parameter-based interpretations like “if the input increases by 1, the output increases by 2” are not that straightforward [47]. Interpretability is crucial when using machine learning models for clinical decision support. Still, authors of cuff-less approaches for BP estimation based on machine learning techniques rarely discuss this topic [36]. One option to increase the interpretability of machine learning models and reduce their training times is using feature selection algorithms, as they enable the model’s parameter reduction [35]. We used three selection feature methods that yielded different optimal subsets with fifteen features each, a relatively small number compared to that for previous work reporting the use of GPR models to estimate BP (see Table 8). Thus, the GPR models presented in this study provide highly accurate BP estimates but also contain a tractable number of features.

**Table 8:** A comparison with previous research on BP estimation using Gaussian process regression models

Author(s), year [Reference]	Dataset	BP ranges (mmHg)	Features	Method (number of features)	Performance
Chowdhury et al., 2020 [22]	Taken from Liang et al., 2018^§^ (126 patients admitted to the Guilin People’s Hospital)	*SBP*: [90–180] *DBP*: [60–100]	Fiducial-based features from PPG, VPG, and APG. Demographic features (height, weight, gender, age, BMI, and HR)	GPR + ReliefF (11 features for SBP; 10 features for DBP)	*SBP*: MAE = 3.02 MSE = 45.49 RMSE = 6.74 R^2^ = 0.90 *DBP*: MAE = 1.74 MSE = 12.89 RMSE = 3.59 R^2^ = 0.92
Nour et al., 2023 [49]	MIMIC III	*SBP*: [82–145] *DBP*: [70–104]	Time-domain and chaotic non-fiducial features	Matern 5/2 GPR for SBP and rational quadratic GPR for DBP (24 features, no feature selection)	*SBP*: MAE = 3.073 MSE = 18.297 RMSE = 4.277 R^2^ = 0.52 *DBP*: MAE = 1.721 MSE = 5.306 RMSE = 2.303 R^2^ = 0.33
Aguet et al., 2023 [18]	Lausanne University Hospital dataset (40 adult patients undergoing general anesthesia)	*SBP*: [83.1–199.5] *DBP*: [42.1–86.4]	Fiducial-based features from PPG, VPG, APG, and JPG. Demographic features (height, weight, age, and gender)	GPR + Least absolute shrinkage and selection operator (Lasso) (35 features for SBP; 24 feat for DBP)	*SBP*: ME = -2.46 STDE = 10.28 *DBP*: ME = -0.84 STDE = 6.88
Shoeibi et al., 2023 [50]	MIMIC II	*SBP*: [92–175] *DBP*: [60–118]	Distances, areas, and perimeters of successive intersection points of Poincaré maps	GPR + *F*-test (73 features)	*SBP*: MAE = 0.79 ME = -0.11 STDE = 3.08 R^2^ = 0.96 *DBP*: MAE = 1.38 ME = -0.0042 STDE = 2.303 R^2^ = 0.88
Present study	Self-collected (47 subjects)	*SBP*: [97–164] *DBP*: [57–109]	Non-fiducial features (PPG, VPG, and APG signal’s mean, skewness, entropy, Kaiser–Teager energy)	Matern 5/2 GPR + ReliefF for SBP and rational quadratic GPR + ReliefF for DBP (15 features)	*SBP*: MAE = 0.44 MSE = 0.61 RMSE = 0.78 ME = -6.44e-14 STDE = 0.035 R^2^= 1 *DBP*: MAE = 0.31 MSE = 0.40 RMSE = 0.63 ME = 4.83 e-14 STDE = 0.033 R^2^= 1

BMI: Body mass index; HR: heart rate; JPG: jerk photoplethysmogram

^§^Available at: https://figshare.com/articles/dataset/PPG-BP_Database_zip/5459299

Differences in how GPR models mapped feature values into BP measurements might also be attributed to kernel selection. Kernels are pivotal functions for machine learning algorithms like GPRs and SVRs. They transform non-linear relationships into linear ones, thus allowing such models to unravel and interpret intricate patterns and relationships [49]. In this sense, linear kernel functions may be enough when relationships between variables are linear. However, non-linear and more complex data might require a polynomial or radial basis function (RBF) kernel. Given that we increased the volume and diversity of training sets by computing features from multiple signal segments, we opted to use RBF kernels to construct GPR models. As shown in Tables 4, 5 and 6, the performance of these algorithms was superior to that of linear regressions and regression trees, which aligns with previous work [50].

Unlike many related works, this study did not find a unique learning model or a single feature subset for predicting SBP and DBP. Instead, our results show that the Matern 5/2 GPR model predicted SBP better than the others, while a rational quadratic GPR outperformed other learning models when estimating DBP. In a previous study [51], the same two models predicted SBP and DBP using one feature subset composed of twenty-four time- and chaotic-domain features with no redundancy elimination (see Table 8). Machine learning models’ performance is feature-dependent [52], so not necessarily one single learning model can perform well when mapping the same feature subset into two different outputs. Furthermore, researchers reporting the same feature subset to predict SBP and DBP separately often find considerable discrepancies between estimation errors [14, 50, 53–55], with one error doubling the other (see Table 9). Even if these differences could not be statistically significant, they highlight an essential (and perhaps overlooked) aspect of BP measurement: the distinction between SBP and DBP. Whereas the former is the pressure the blood exerts against the inner arterial walls when the heart beats, the latter represents the pressure exerted between heartbeats [56]. As these measurements reflect the various phases of the cardiac cycle, it is reasonable to expect that the most relevant PPG-based features for SBP estimation may not hold the same significance for DBP prediction, and vice versa. This is our rationale for employing feature selection techniques for each target variable. Let us consider the top 15 features chosen by ReliefF (see Table 3). Although SBP and DBP share certain features, entropies are more pertinent for SBP estimation, while energy variances are more valuable for estimating DBP. Consequently, these differences likely led to the development of two distinct models to predict SBP and DBP separately. In this regard, it is essential to understand what each measurement truly signifies, and which tool is most effective for assessing it, rather than relying on a single method to predict two separate values.

**Table 9:** Performance of several PPG-based approaches using the same feature subset and learning model for SBP and DBP Estimation

Author(s), year [Reference]	Learning model and Feature subset	MAE (mean ± standard deviation)	
		SBP	DBP
Liu et al., 2017 [14]	SVR + 35 time-domain and morphological features from PPG and APG	8.54 ± 10.9	4.34 ± 5.8
Kachuee et al., 2016 [52]	AdaBoost + 15 Physiological and time-domain indexes derived from PPG and ECG signals	11.17 ± 10.09	5.35 ± 6.14
Hasanzadeh et al., 2019 [54]	AdaBoost + 14 fiducial-based features from PPG signals	8.22 ± 10.38	4.17 ± 4.22
Zhang et al., 2017 [55]	SVR + 9 morphological features of the PPG signal	11.64 ± 8.20	7.61 ± 6.78
Khodabakhshi et al., 2022 [56]	Parallel deep convolutional neural network + 36 features extracted from Poincaré plots, recurrence quantification	1.73 ± 2.76	3.81 ± 6.13

One of the advantages of using non-fiducial features is not requiring precise detection of points on the signal for extracting valuable features. On the other hand, this is not the first study employing non-fiducial features of the PPG signal for BP estimation. For example, Monte-Moreno and co-workers [27] used features like the zero crossing rate (ZCR), the spectral entropy, and the Kaiser–Teager energy (KTE) to train several machine-learning models, among which the random forest algorithm attained the highest coefficients of determination (*r*^2^ = 0.91 and 0.89 for SBP and DBP, respectively) and the grade B according to the British Hypertension Society (BHS) criteria [57]. A more recent study by Shoeibi and colleagues [58] reports the usefulness of several features extracted from Poincaré plots [34] for accurate BP estimation (see Table 8), with which they satisfied both AAMI [59] and BHS standards. Features included distances, areas, and perimeters of successive intersection points of Poincaré maps computed from a 2-D phase-space reconstruction of PPG signals.

Interestingly, non-fiducial features have shown not to be as relevant when combined with other domains’ features (e.g., time, frequency, chaos). Yao and colleagues combined four sets of features, among which statistical indexes, such as signal variance, kurtosis, and skewness, were included [25]. However, only the signal variance showed a moderate correlation with the target variables (*r* = 0.43 and 0.36 for SBP and DBP, respectively), and the most relevant feature subsets were predominantly composed of morphological and demographic data. In another study [26], deep learning models were combined with time-, frequency-, and statistics-domain features to provide accurate SBP and DBP estimation. The authors found that gated recurrent units (GRU) and bidirectional long short-term memory (Bi-LSTM) networks trained with time-domain features performed better than other models, including random forests and SVMs. It is worth mentioning that the abovementioned research groups considered non-fiducial features from the PPG signal only and did not include information extracted from its derivatives. Instead, we took account of the potential contribution of a broader set of non-fiducial features by including VPG and APG waveforms, and we explored several other features not previously reported in the literature (see Table 2). As shown in Table 3, PPG, VPG, and APG signals’ skewness and interquartile ranges of energy operators can be relevant for BP estimation, thus confirming previous results [27].

### Limitations

Despite its promising results, this study comes with several limitations. The first one is related to the modest sample size we used and its narrow span of BP measurements. We could not compare our method to the international AAMI standard because we did not meet the requirement of including at least 85 subjects [59]. However, the potential influence of various pathophysiological conditions and medications on PPG waveforms was partially controlled, as we did not recruit hospitalized individuals. A recent study indicated that data from patients with critical health conditions, often under pharmacological treatment (e.g., MIMIC), may exhibit a skewed relationship between BP and several PPG features, thus hindering the generalization of BP estimation algorithms [17]. Our dataset poses a helpful contribution to addressing the need for appropriate and objective validation of BP estimation models on non-hospitalized populations. It includes subjects from the four BP categories established by the American Heart Association [30] and is publicly available via GitHub (link: https://github.com/sanvsquezsz/PPG-based-BP-assessment). Another limitation of this work is that we could have overlooked non-fiducial features potentially meaningful for BP estimation. Spectral [28, 60] and chaotic features [51] extracted from PPG signals hold some promise for developing cuff-less BP measurement methods. Still, they are not as easy to calculate as time-domain and statistical features, thus becoming prohibitive for computationally-restrained hardware. Keeping in mind that there is an interest in developing lightweight methods for continuous BP monitoring, we included a set of features that are relatively easy to compute. As PPG-based features may not be enough for predicting BP accurately [61], non-fiducial feature extraction was extended to include the first and second derivatives of the PPG signal, with results confirming the relevance of these waveforms to the context of BP estimation. Third, we employed a locally designed data acquisition system, so utilizing third-party equipment may considerably alter the findings. Nevertheless, we selected an option that has proven valuable in previous studies [62, 63]. Based on its cost-effectiveness, power consumption, and easy-to-deploy character, this option aligns with developing a portable and affordable alternative for BP estimation. The system we used also provides advantages in terms of scalability, given its microprocessor-based design.

The fourth limitation is that our results, in their current form, may not reveal their clinical relevance as straightforwardly as expected. Error metrics (MSE, RMSE…) provide an objective framework for benchmarking machine learning models. However, they have rarely been translated into clinical settings, so clinicians may find the capabilities of the regression models to deliver medically relevant outcomes hard to appreciate. One alternative to this issue is to promote knowledge exchange and collaboration between physicians and computational science researchers [64]. In this sense, it is crucial to materialize a shared understanding of the other’s domain to develop more clinically relevant machine learning models. Finally, we did not perform a statistical significance test to compare the regression models’ performance. Given the great variety of regression models used (17 in total), each trained and tested on three different optimal feature subsets, the multiple-testing problem task would be highly complicated, thus making classical statistical inference less tractable due to the large number of comparisons we should perform [65]. According to several authors [66, 67], statistical tests should be applied cautiously in machine learning studies as the exclusive focus on rejecting null hypotheses may contribute to misunderstanding the experiments’ results. The present work is an exploratory study, so we prioritized its readability and logical flow over statistical inference, which is more appropriate when developing project-specific probability models.

## Conclusion

There has been intensive research in developing and testing machine learning-based approaches for BP estimation using features extracted from the PPG signal and its derivatives. While many of them relying on fiducial point detection hold promising results, they are subject to the accuracy and robustness of the method used for identifying those landmarks. This study aimed to provide an alternative for BP estimation using non-fiducial features from PPG, VPG, and APG waveforms and supervised machine learning algorithms. We found different optimal feature subsets for SBP and DBP via three selection techniques, with the Matern 5/2 and the rational quadratic GPR models providing, respectively, the lowest prediction errors when combined with ReliefF. The proposed approach employs only fifteen features, many of which are relatively easy to compute (e.g., signal skewness), thus becoming suitable for embedded applications. Still, this method requires further validation due to the limited sample size, so we intend to continue optimizing it as more data becomes available. We recommend paying attention to the PPG features selected for predicting SBP and DBP, as these measurements are recorded during different phases of the cardiac cycle. In the meantime, forthcoming research on PPG-based methods for predicting BP may benefit from the dataset we made publicly available.
